# Diet, Ayurveda and interface with biomedicine

**DOI:** 10.4103/0975-9476.74423

**Published:** 2010

**Authors:** A Chopra, M Saluja, G Tillu

**Affiliations:** *Center for Rheumatic Diseases, Hermes Elegance, 1988 Convent St., Camp, Pune-411008, India. E-mail: crdp@vsnl.net*

We thank Dr Kontum for his interesting and useful comments to our paper[1] and further reference to the address by one of the authors (AC) to the recently concluded World Ayurveda Congress 2010 in Bangalore on the subject of ‘Ayurveda Biomedicine Interface’. Kontum has very rightly highlighted the ‘holistic approach’ as a contrast to just focusing on ‘drugs’. Also, he has described a relatively simple method of cooking rice practiced by his family that apparently enriches the health promotion and preventive value. Several such healthy practices advocated by Ayurveda could be included in the daily life style and living in modern times in addition to the one he suggested.

Diet and lifestyle practices pose interesting questions for researchers. Causative and therapeutic effects of various dietary recipes have been elaborately described in Ayurveda classics, especially for Rheumatoid Arthritis. Though general diet-lifestyle guidelines of Ayurveda emphasize consuming fresh, hot and easy to digest food, several variations are seen as customs and traditions. Various kinds of variable – social, geographic and climatic – may determine the choice of variant. Ayurveda recommends diet considering them all. For example, in coastal areas, food items prepared by fermentation procedures are common, though this is not the case in other regions. Hence effects of diet and lifestyle may be very specific. Sometimes recommended diet is complex and patient compliance is low. To ensure compliance, diet should be balanced, practical and easy to follow.

Efficacy of treatment packages needs to be studied systematically. In such situations, observational study designs should be adopted. We recommend the case series approach with proper documentation, including medical and family history, details of diagnosis, investigations and ongoing treatment. Very often, such meticulous observations can result in interesting research leads.

Globalization and related developments in the modern World are adding to available dietary recipes; some may be causative agents, others possible additions to therapeutic armament. Careful observations like those mentioned by Kontum are useful to explore wisdom from ancient traditions and should be taken further as specific research questions. Ayurveda itself evolved and developed through such methodical observations.

Validating the whole of Ayurvedic therapy from a modern bioscience perspective presents a difficult task. However, keeping in tune with modern science and the times (modern age and man), Ayurveda may benefit from allowing itself to be modernized. After all, its aim is to serve mankind by going global. Our experiments on Ayurvedic drugs demonstrating their therapeutic usefulness represent a useful start. Our results from earlier trials differed from expectations based on Ayurvedic practice and classic literature. They suggested that Ayurvedic drugs need to be augmented.

We are aware that some of that failure could be due to our controlled drug trials missing out critical components of Ayurveda’s therapeutic approach like diet and lifestyle. However, it was a learning curve for all of us in the Ayurveda-Modern Medicine group. We have since moved ahead. The NMITLI arthritis project was a team India effort that taught us several lessons about Ayurvedic drug development.[2] As a result, improved methods of validation have been defined and are now being researched. We strongly believe that in several difficult to treat disorders (like arthritis) ‘Ayurvedic care packages’ ought to be standardized and evaluated in controlled clinical trials largely based on Ayurvedic diagnosis, including prakriti, and monitored for drug response and safety. Trial designs and analysis must be specific to Ayurveda. As long as resources and funds remain scarce, they should be spent on strengthening clinical Ayurveda, rather than on fundamental research issues like structure-activity relationships, or mechanism of action. Endorsing safety through realistic clinical methods should be of paramount priority.[3]

Above all, there is a need to interface or integrate important clinical therapy components from Ayurveda and modern medicine for better treatment outcomes. Ayurveda and its practitioners need to actively participate in meeting this challenge.
